# Modeling Dominant Macrobenthic Species Distribution and Predicting Potential Habitats in the Yellow River Estuary, China

**DOI:** 10.3390/biology14121732

**Published:** 2025-12-03

**Authors:** Chao Yuan, Juan Huang, Lan Wang, Tao Zhang, Haolin Yu, Huiying Sun, Yumeng Liu, Shuo Sun, Jingyi Sun, Yongjun Shang, Jie Feng, Jiangling Xu

**Affiliations:** 1 North China Sea Marine Forecasting and Hazard Mitigation Center, Ministry of Natural Resources, Qingdao 266061, China; 2 Key Laboratory of Ecological Prewarning and Protection of Bohai Sea, Ministry of Natural Resources, Qingdao 266033, China; 3 CAS Key Laboratory of Marine Ecology and Environmental Sciences, Institute of Oceanology, Chinese Academy of Sciences, Qingdao 266400, China; 4 Center for Ocean Mega-Science, Chinese Academy of Sciences, Qingdao 266400, China; 5 Fisheries College, Ocean University of China, Qingdao 266003, China

**Keywords:** macrobenthos, species distribution, potential habitats, Yellow River Estuary, boosted regression trees

## Abstract

Macrobenthic fauna are vital to the ecological health of the Yellow River Estuary, yet their long-term population drivers are poorly understood. This study used Boosted Regression Tree models to analyze the spatio-temporal distribution of five dominant species: *Glycinde gurjanovae*, *Sternaspis scutata*, *Moerella jedoensis*, *Theora fragilis*, and *Raphidopus ciliatus*. Key environmental drivers included ammonia nitrogen, water depth, sand content of sediment, and water temperature. Specifically, *S. scutata* and *R. ciliatus* preferred deeper waters, *M. jedoensis* favored habitats with moderate sand content of sediment, *T. fragilis* primarily occurred at water temperatures lower than 25 °C, and *G. gurjanovae* distribution was most influenced by ammonia nitrogen. All species exhibited a preference for lower ammonia nitrogen concentrations. Inorganic nitrogen and freshwater discharge from the Yellow River significantly influenced the distribution of *G. gurjanovae*, whereas river discharge alone was positively correlated with areas exhibiting a high occurrence probability (>0.5) for *M. jedoensis*. Future studies that integrate comprehensive seasonal monitoring data, hydrodynamic conditions, and food availability could further enhance predictive accuracy, providing stronger theoretical and technical support for ecological conservation and management in the Yellow River Estuary.

## Simple Summary

Estuarine ecosystems, where rivers meet the sea, are among the most productive and biologically rich environments on Earth, but they face growing threats from human activities and climate change. This study focuses on the Yellow River Estuary in China—a vital wetland area that supports many marine species. We aimed to understand how key bottom-dwelling animals (like worms, clams, and crustaceans) respond to environmental changes here based on long-term data. Using a computer-based prediction model, we found that water depth and nutrients such as ammonia nitrogen strongly influence where these species can live. Importantly, reducing ammonia nitrogen input from the Yellow River could significantly improve habitat quality for these organisms. Our results provide a scientific basis for better managing and protecting this ecologically and economically important region, ensuring it remains healthy for future generations.

## 1. Introduction

The spatial distribution of species is a key determinant of ecosystem structure and function. It influences community composition and trophic dynamics, playing a crucial role in maintaining ecosystem stability and supporting species coexistence [1,2,3]. In the context of global climate warming, changes in species distributions have garnered increasing attention, as rising temperatures drive many species poleward and upward, causing contractions in their original ranges [4,5,6]. Simultaneously, anthropogenic disturbances, such as habitat destruction and environmental pollution, have altered species distributions and, in some cases, may have even led to the local extirpation of certain species [7,8]. These shifts undermine ecosystem stability and functions, thereby accelerating biodiversity loss. Understanding species distribution patterns and their driving factors is essential for predicting species responses to environmental change and developing effective management and conservation strategies.

Estuarine ecosystems, located in the transition zone between land and sea, are biodiversity hotspots with high primary productivity and complex habitat gradients. These ecosystems play a crucial role in maintaining biodiversity and ecosystem stability, yet they are highly vulnerable to multiple stressors, such as habitat degradation, pollution, and climate change [9]. The Yellow River Estuary, located in Bohai Sea, China, is the largest newly formed estuarine wetland ecosystem in the warm temperate zone [10]. The estuarine ecosystem is strongly shaped by substantial freshwater input, sediment deposition, and nutrient fluxes from the Yellow River [11,12]. It supports essential ecological functions, providing crucial habitats for spawning, foraging, overwintering, and migration of various marine species [13,14].

Macrobenthic fauna represent a key functional group in estuarine and coastal ecosystems. They engage in feeding, bioturbation, and burrowing, thereby altering sediment structure and regulating nutrient dynamics [15]. By consuming plankton, benthic algae, and organic detritus, they facilitate nutrient cycling. At the same time, they serve as prey for fish and crustaceans and play an essential role in energy transfer and material cycling in marine ecosystems [16,17]. Due to their long life cycles, limited mobility, and high sensitivity to environmental changes, macrobenthic species are widely recognized as reliable bioindicators of marine ecosystem health [12]. A comprehensive understanding of their distribution patterns and habitat suitability is essential for effective marine ecosystem conservation and resource management.

The macrobenthic communities in the Yellow River Estuary have been extensively studied for decades. As early as the mid-1980s, Zhang et al. (1990) conducted comprehensive surveys of species composition, abundance, and biomass [18]. In recent years, research has broadened to encompass the structural characteristics, spatio-temporal distribution, and environmental drivers of macrobenthic communities in intertidal and subtidal zones [16,19,20,21,22]. Additionally, studies have also investigated how anthropogenic activities, such as water diversion and sediment regulation, influence macrobenthic communities [23,24,25]. However, existing studies exhibit several notable limitations: (1) discontinuous temporal coverage, with insufficient continuous observational data to support long-term trend analyses; (2) a lack of in-depth analysis of environmental driving mechanisms, with a limited ability to quantify nonlinear relationships between multiple environmental factors and species distributions; and (3) underdeveloped predictive modeling approaches, hindering the accurate forecasting of spatio-temporal distribution dynamics. These gaps hinder a comprehensive assessment of distribution patterns, historical trends, and projected changes in macrobenthic communities in the Yellow River Estuary.

Machine learning algorithms, recognized for their ability to model complex nonlinear relationships [26], have been widely applied in marine species distribution modeling [27]. However, their application in predicting macrobenthic species distributions remains limited [17]. The boosted regression trees (BRT) machine learning algorithm, which integrates decision trees with boosting techniques, iteratively refines model performance to improve predictive accuracy [28]. Compared with traditional regression models, BRT demonstrates superior robustness in capturing complex nonlinear relationships and handling outliers, rendering it particularly effective for ecological modeling. Several studies have suggested an outperformance of BRT over traditional regression models in species–habitat relationships [28,29,30,31,32].

This study aims to systematically examine the spatio-temporal dynamics of dominant macrobenthic species in the Yellow River Estuary over the past years, assess their environmental interactions, and forecast their future distributions. To achieve these objectives, the BRT machine learning approach was used to develop species distribution models. Using this modeling framework, we analyze the responses of macrobenthic species to estuarine environmental changes, identify key environmental drivers shaping their distributions, and reconstruct their spatio-temporal trends over the past two decades. This research aims to enhance the understanding of macrobenthic species distribution patterns and their environmental drivers in the Yellow River Estuary, providing critical insights and technical support for ecosystem conservation and resource management.

## 2. Materials and Methods

### 2.1. Field Survey and Data Collection

**Figure 1:**
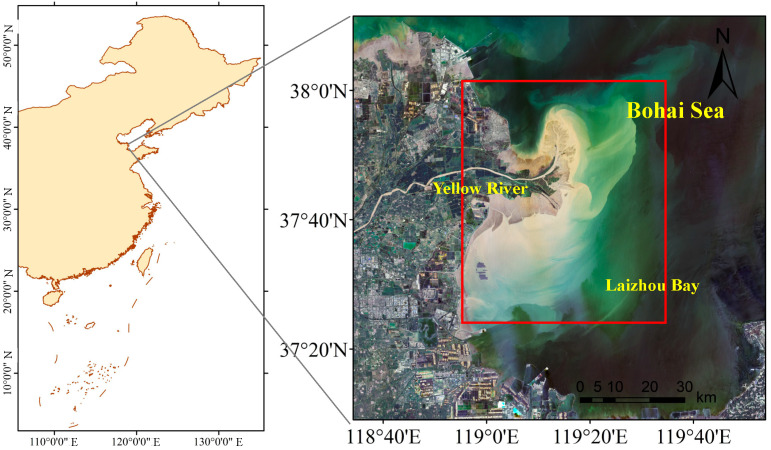
Study area in the Yellow River Estuary, China. Red rectangle in the left indicates the sampling area, the satellite image is acquired by Landsat 9 on 28 July 2025.

This study uses data from historical summer surveys of environmental and biological resources around the Yellow River Estuary, China (Figure 1). During each field survey, macrobenthic organisms, water, and sediment samples were collected from every survey station and transported to the laboratory for further analysis: macrobenthic samples were obtained using a grab sampler (0.05 m^2^ sampling area), with 3–5 replicates taken at each station, and the collected samples were then sieved through a 0.5 mm mesh to retain benthic organisms and remove sediment particles. All macrobenthic samples were fixed with formalin and delivered to the laboratory. Species identification were conducted under a stereomicroscope. For water quality assessment, in situ measurements were conducted for water depth, temperature, pH, dissolved oxygen, salinity with a calibrated YSI multi-parameter water quality probe, while water samples were also collected and transported to the laboratory to analyze chlorophyll-a (chl-a), reactive phosphate, nitrite nitrogen, ammonia nitrogen, nitrate nitrogen, and petroleum hydrocarbons; sediment surveys focused on grain size analysis and organic carbon content determination, and all sample collection and measurement procedures followed the protocols outlined in China’s national standards for marine surveys [33,34,35] (GB/T 12763.4-2007; GB/T 12763.6-2007; GB/T 12763.9-2007).

### 2.2. Analysis of Dominant Macrobenthic Species

The Index of relative importance (IRI) [36], was used to quantify and identify the dominant macrobenthic species. In each summer survey, the five species with the highest IRI values were classified as dominant species. The IRI was calculated as follows:

(1) $$Y=(n_{i}/N)\times f_{i}$$

where N is the total number of macrobenthic species recorded in each survey, $n_{i}$ represents the abundance of the macrobenthic species indexed by *i*, and $f_{i}$ is its frequency of occurrence in the survey.

### 2.3. Redundancy Analysis Between Dominant Species and Environmental Variables

This study employed distance-based redundancy analysis (db-RDA) to investigate the constraining effects of environmental variables on benthic community structure. The species presence–absence data underwent Hellinger pretreatment, while environmental factors were standardized to eliminate dimensional influences. The db-RDA model was subsequently executed, with the significance of both the overall model and individual environmental factors assessed through 999 permutation tests. Collinearity among environmental variables was assessed via the Variance Inflation Factor (VIF), with variables showing VIF > 4 being progressively excluded [37]. Finally, the relationships between species and environmental factors within the ordination space were visualized through a biplot, with key environmental drivers interpreted based on the explanatory variance of constrained axes and factor loading strengths.

### 2.4. Construction of Species Distribution Models Using BRT

#### 2.4.1. Introduction to the BRT Model

BRT is an adaptive ensemble learning method that combines boosting with classification and regression tree (CART) [32,38,39]. Unlike traditional regression techniques, BRT is not designed to identify a single optimal model. Instead, it enhances model performance by aggregating an ensemble of shallow decision trees [39]. A BRT model can be formulated as a weighted sum of M decision trees:

(2) $$f_{M}\left(x\right)=\sum_{m=1}^{M}T_{m}(X,\gamma_{m})$$

where X denotes the set of predictor variables, $T_{m}(X,\gamma_{m})$ is the m-th decision tree, and $\gamma_{m}$ defines the tree’s split points and leaf node values. The process of optimizing $\gamma_{m}$ corresponds to the learning procedure of an individual decision tree.

#### 2.4.2. Selection of Model Parameters

BRTs are trained in a sequential, iterative manner, where each decision tree is added to improve model performance [40]. The learning process of BRT is governed by three key parameters. First, during training, each decision tree is built using a randomly sampled subset of the observations, known as the bagging fraction, which introduces randomness to enhance model predictive accuracy [41]. Second, to control the learning rate and mitigate overfitting, each new tree is scaled by a small shrinkage factor (v, typically <0.1) before being added to the model. This factor is commonly referred to as the learning rate (*lr*). Finally, all decision trees in a BRT model have a fixed number of leaf nodes. Once a tree reaches the specified number of terminal nodes, it stops growing, eliminating the need for pruning. The number of leaf nodes per tree, known as tree complexity (*tc*), determines the order of interactions that the model can capture among predictor variables [40]. Among these three parameters, the recommended bagging fraction typically ranges from 0.50 to 0.75 [39]. The values of *lr* and *tc* have a greater impact on model performance. Therefore, *lr* is tested at 0.001, 0.005, 0.01, and 0.1, while *tc* is set at 1, 3, 5, 8, and 15. To assess model performance, 90% of the data is randomly selected for model training, while the remaining 10% is reserved for evaluating prediction error. The optimal values of *lr* and *tc* are selected based on model fit and prediction accuracy.

#### 2.4.3. Optimal Number of Decision Trees

As the number of decision trees increases, the BRT model achieves a better fit to the training data. However, an excessive number of trees may lead to overfitting, thereby compromising the model’s predictive performance. The optimal number of trees is identified by minimizing the mean prediction error. A final species distribution model is trained using the entire dataset with the optimal number of decision trees.

#### 2.4.4. Calculation of Predictor Importance

For an individual CART, the importance of a predictor variable Xn in relation to the response variable is measured by Jn(T) [32], where

(3) $$J_{n}^{2}\left(T\right)=\sum_{t=1}^{tc-1}j_{n}^{2}I\left(v\left(t\right)\right)=n,$$

where T represents a CART with *lc* leaf nodes, and $j_{n}^{2}I\left(v\left(t\right)\right)=n$ denotes the reduction in squared error risk when Xn is selected as the splitting variable at internal node t. In a BRT model, the relative importance of a predictor Xn, denoted as $J_{n}^{2}$, is computed as the average reduction in squared error risk across all M classification and regression trees in the model [40,41]:

(4) $$J_{n}^{2}=\frac{1}{M}\sum_{M=1}^{M}J_{n}^{2}(T_{m}).\;$$

in practice, the results are typically normalized so that the sum of the relative importance values for all predictors equals 1.0. The importance of each predictor is then expressed as a percentage.

#### 2.4.5. Performance Evaluation and Validation of the Predictive Model

Model Accuracy Evaluation: The environmental and spatio-temporal variables from the validation dataset are incorporated into the predictive model to estimate the corresponding occurrence probabilities. Model performance is assessed based on predictive deviance, the area under the receiver operating characteristic curve (AUC), root mean squared error (RMSE), and mean absolute error (MAE) [42]. Predictive deviance is computed as follows [43]:

(5) $$Dev=-2\frac{1}{\sum w_{i}}\sum w_{i}\left[y_{i}f\left(x_{i}\right)-\log\left(1+e^{f\left(x_{i}\right)}\right)\right]$$

in this equation, (x, y) denotes a test data point, and w represents the assigned weight for each test sample, with all test samples assigned a uniform weight of 1.0. The function f(x) corresponds to the raw output of the BRT model.

Model Performance Evaluation: The performance of the optimized model was evaluated using 10-fold cross-validation. The RMSE was computed to assess the difference between predicted values and observed values. Additionally, a linear regression was performed between predicted values ($P_{i}$) and observed values ($O_{i}$) to derive the slope, intercept, and coefficient of determination (R^2^), which serve as key performance metrics. This process was repeated 100 times to ensure robustness. The RMSE was calculated using the following equation [44]:

(6) $$RMSE=\sqrt{\frac{1}{n}\sum_{i=1}^{n}{\left(O_{i}-P_{i}\right)}^{2}}$$

simultaneously, the MAE was also computed as an important validation parameter. The MAE quantifies the absolute difference between predicted and actual values for each data point and then averages these absolute errors. The MAE was calculated as follows:

(7) $$MAE=\frac{1}{n}\sum_{i=1}^{n}\left|y_{i}-y_{p}\right|$$

where n represents the number of data points, $y_{i}$ is the actual target value (ground truth), and $y_{p}$ is the model’s predicted value for each data point. One key advantage of MAE is its robustness to outliers, making it a valuable metric for evaluating model performance on datasets containing extreme values.

All model development and validation procedures were implemented in R 4.4.0 using the “vegan” (version 2.7-2) and “gbm” (version 2.2.2) packages.

### 2.5. Spatio-Temporal Patterns of Occurrence Probability of Dominant Macrobenthic Species

To characterize the spatio-temporal distribution patterns of dominant macrobenthic species in the Yellow River Estuary, the first three dominant species were selected as target species. BRT models were used to estimate the occurrence probability of these species at each survey station in August of each year. The predicted probabilities were interpolated via the inverse distance weighting method at a spatial resolution of 0.01 degrees, producing a continuous spatial distribution of occurrence probability across the Yellow River Estuary.

Predicted probabilities were categorized into two ranges: 0.0–0.5 and 0.5–1.0. Regions with probabilities between 0.0 and 0.5 were identified as low-occurrence probability zones, while regions with probabilities between 0.5 and 1.0 were designated as high-occurrence probability zones. To assess the impact of annual Yellow River discharge on the spatial extent of low-occurrence probability zones (0.0–0.5), the Majorize-Minimize Robust Regression Estimation Method (MM Robust Regression) was applied. This method first utilizes S-Estimator of Regression (S-estimation) or Least Trimmed Squares Estimation to obtain initial parameter estimates, followed by optimization using iteratively reweighted least squares. The weight adjustment is performed using M-estimation loss functions, enhancing both estimation efficiency and robustness. A key advantage of this approach is its resilience to outliers, making it particularly effective in improving model robustness and predictive performance [45,46]. All analyses were conducted in R (version 4.4.0) using the “gstat”(version 2.1-4) package for spatial interpolation and the “robustbase” (version 0.99-6) package for robust regression analysis.

## 3. Results

### 3.1. Dominant Macrobenthic Species

Dominant macrobenthic species in the Yellow River Estuary were identified. The most frequently occurring dominant species were sequentially listed as *Glycinde gurjanovae* (Uschakov & Wu, 1962), *Sternaspis scutata* (Ranzani, 1817), *Moerella jedoensis* (Lischke, 1872), *Theora fragilis* (A. Adams, 1856), *Raphidopus ciliatus* (Stimpson, 1858), *Heteromastus filiformis* (Claparède, 1864), *Protankyra bidentata* (Woodward & Barrett, 1858), *Laternula marilina* (Reeve, 1860), and others. No invasive species was recorded. Based on these findings, the first five representative species were selected for the subsequent development of species distribution models to analyze the spatial distribution patterns of macrobenthic organisms.

### 3.2. Relationships Between Dominant Macrobenthic Species Distribution and Environmental Variables

The environmental variables used to analyze the relationships with the dominant macrobenthic species distribution include water depth, pH, dissolved oxygen, salinity, reactive phosphate, ammonia nitrogen, nitrate nitrogen, nitrite nitrogen, chl-a, sand content of sediment, silt content of sediment, and nitrogen-to-phosphorus ratio. An assessment of collinearity among these variables, based on VIFs, indicated that all values range between 1.20 and 1.90, which falls within an acceptable range for the RDA analysis and following model construction using the BRT method (Table S1).

**Figure 2:**
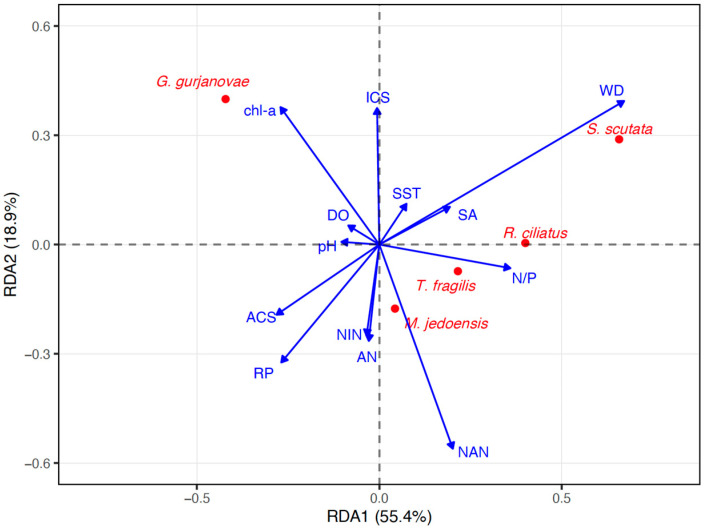
Biplot between dominant macrobenthic species and environmental factors from RDA analysis.

The RDA analysis results show that the first two constrained axes cumulatively account for approximately 74.3% of the constrained variance (Figure 2), with the first axis serving as the primary gradient axis. The envfit test identified six significant environmental determinants (*p* < 0.05): water depth, nitrate nitrogen, phosphate, nitrogen-to-phosphorus ratio, chlorophyll-a, and sand content of sediment. Figure 2 shows an approximately perpendicular distribution between water depth and nutrient factors, revealing that the water depth gradient and the nutrient gradient together constitute a two-dimensional ecological space for the niche differentiation among different species. *R. ciliates* and *S. scutata* show a significant positive correlation with water depth, indicating their preference for deepwater environments; *G. gurjanovae* and *T. fragilis* are closely associated with nutrient factors such as nitrate nitrogen and chl-a, reflecting their adaptation to eutrophic waters; while *M. jedoensisis* distributed near the origin, suggesting a relatively weak response to the illustrated environmental factors.

### 3.3. BRT Modeling of Dominant Macrobenthic Species Distribution

Various combinations of predictor variables were evaluated during the BRT model training, and predictor variables were retained if the model achieved an AUC ≥ 0.7. The selected variables were then used to develop species distribution models for each target species. The environmental variables selected for the final species distribution models are listed in Table 1.

#### 3.3.1. *G. gurjanovae*

**Figure 3:**
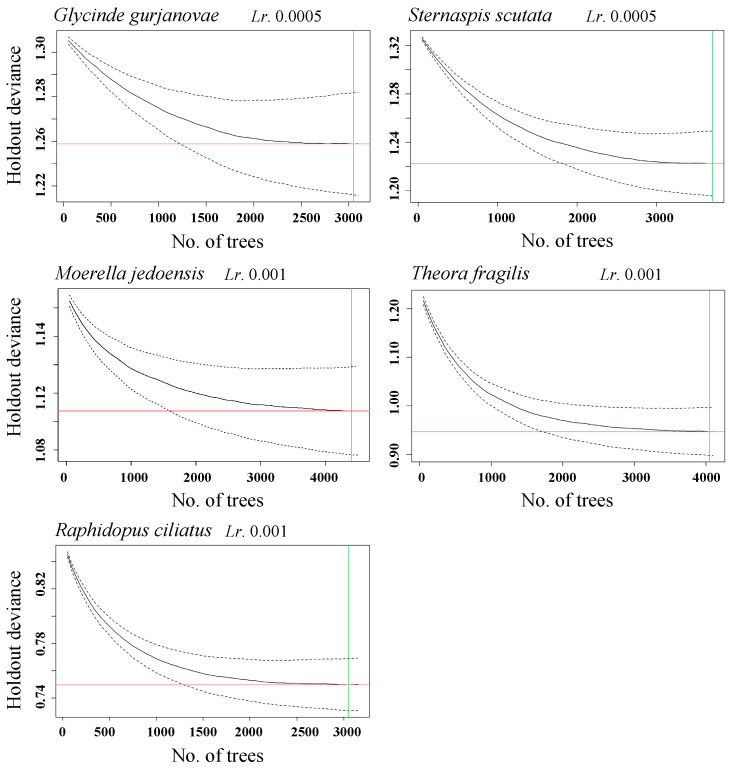
Relationship between the number of decision trees and model prediction bias under varying learning rates and model complexities. The solid line shows the average model performance, the dashed lines show the performance variability, and the red line indicates the minimum number of trees required for the model to reach a stable state.

**Figure 4:**
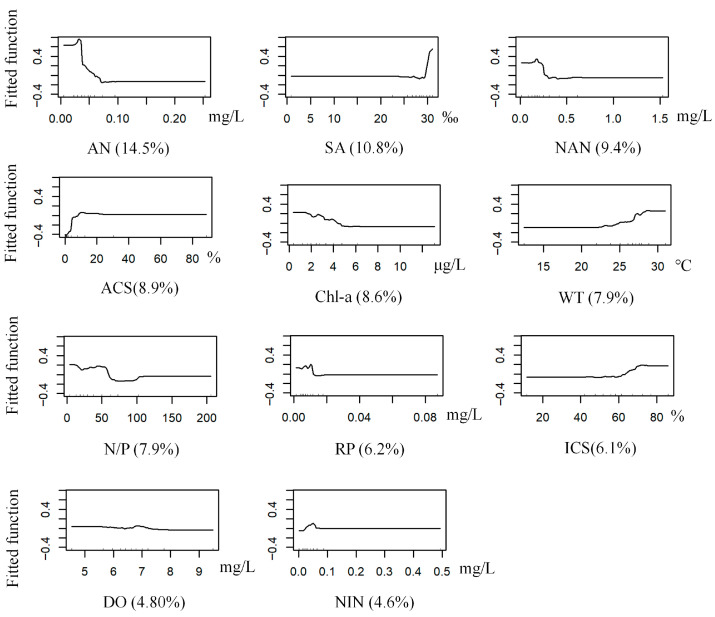
Response curves of *G. gurjanovae* occurrence probability to environmental variables (Abbreviations as in Table 1).

The BRT model for *G. gurjanovae* achieved optimal performance with a learning rate of 0.0005, a bagging fraction of 0.5, a tree complexity of 5, and 1800 trees. The model had a mean deviance of 1.18, RMSE 0.35, training AUC 0.96, and cross-validation AUC 0.73. Ammonia nitrogen was the most influential predictor (14.50% contribution to the model), followed by salinity (10.80%). The predicted probability of occurrence was relatively high within the ammonia nitrogen range of 0.25–0.30 mg/L. It was also elevated at a salinity of approximately 30‰. (Table 2 and Table 3, Figure 3 and Figure 4).

#### 3.3.2. *S. scutate*

**Figure 5:**
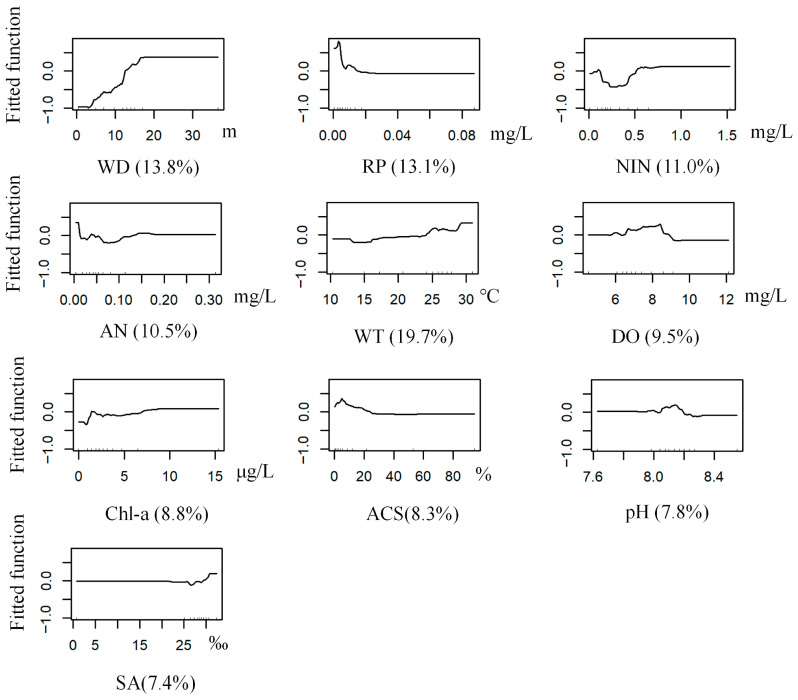
Response curves of *S. scutata* occurrence probability to environmental variables (Abbreviations as in Table 1).

The BRT model for *S. scutata* achieved optimal performance with a learning rate of 0.005, a bagging fraction of 0.5, a tree complexity of 15, and 3700 trees. The model had a mean deviance of 1.11, RMSE 0.34, training AUC 0.97, and cross-validation AUC 0.75. Water depth was the most influential predictor (13.81% contribution to the model), followed by reactive phosphate (13.80%), nitrate nitrogen (13.10%), and ammonia nitrogen (11.10%). The predicted probability of occurrence increased with water depth up to 17 m and remained high at depths ≥ 17 m. Additionally, *S. scutata* had a higher predicted probability of occurrence when reactive phosphate was approximately 0.003 mg/L and nitrate nitrogen exceeded 0.6 mg/L (Table 2 and Table 3, Figure 3 and Figure 5).

#### 3.3.3. *M. jedoensis*

**Figure 6:**
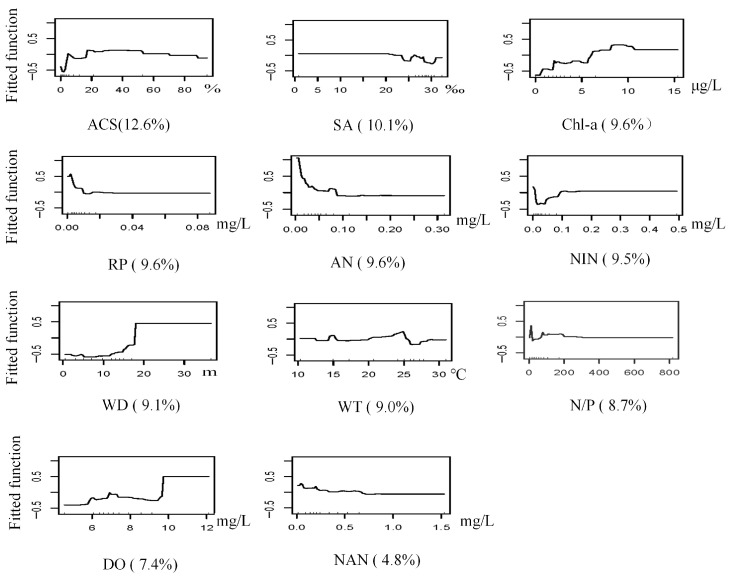
Response curves of *M. jedoensis* occurrence probability to environmental variables (Abbreviations as in Table 1).

The BRT model for *M. jedoensis* achieved optimal performance with a learning rate of 0.001, a bagging fraction of 0.5, a tree complexity of 5, and 4400 trees. The model had a mean deviance of 1.19, RMSE 0.36, training AUC 0.96, and cross-validation AUC 0.71. Sand content of sediment was the most influential predictor (12.60% contribution to the model), followed by salinity (10.13%) and chl-a (9.6%). The predicted probability of occurrence was highest when sand content ranged from 19% to 54%. Additionally, increasing chl-a concentrations was associated with a higher predicted probability of occurrence, with a peak at approximately 10 µg/L (Table 2 and Table 3, Figure 3 and Figure 6).

#### 3.3.4. *T. fragilis*

**Figure 7:**
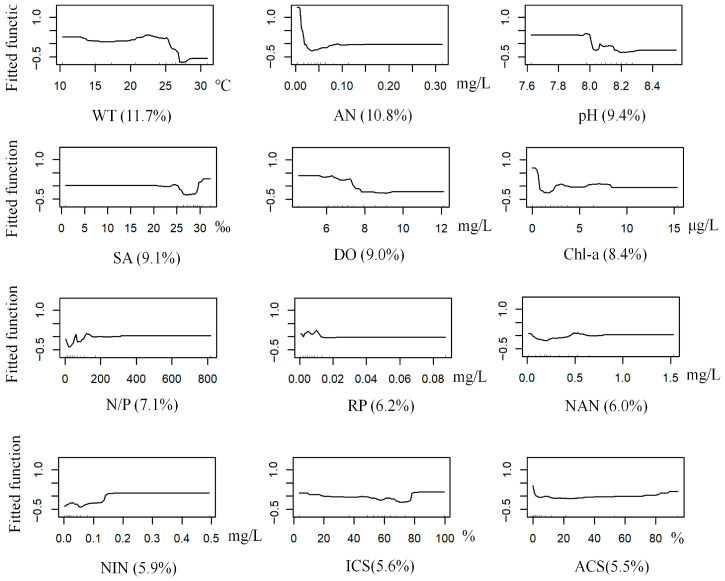
Response curves of *T. fragilis* occurrence probability to environmental variables (Abbreviations as in Table 1).

The BRT model for *T. fragilis* achieved optimal performance with a learning rate of 0.001, a bagging fraction of 0.5, a tree complexity of 5, and 4020 trees. The model had a mean deviance of 0.91, RMSE 0.25, training AUC 0.99, and cross-validation AUC 0.78. Water temperature was the most influential predictor (11.70% contribution to the model), followed by ammonia nitrogen (10.80%). The highest predicted probability of occurrence was observed at approximately 23 °C, while the probability remained high when ammonia nitrogen was <0.1 mg/L (Table 2 and Table 3, Figure 3 and Figure 7).

#### 3.3.5. *R. ciliates*

**Figure 8:**
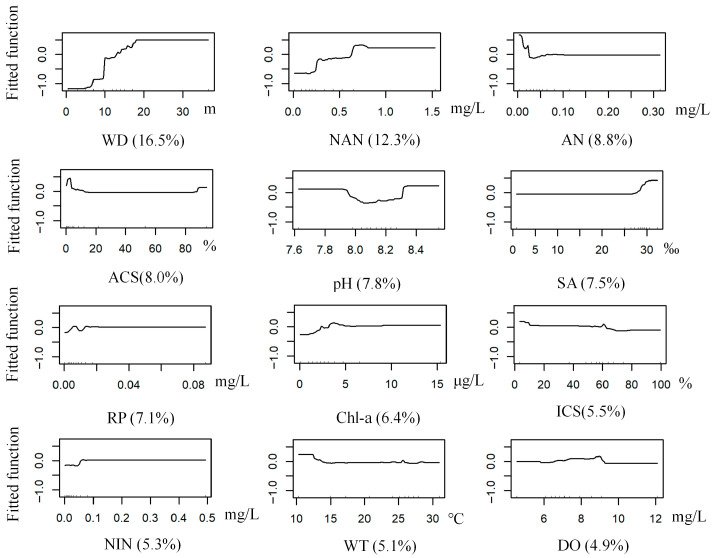
Response curves of *R. ciliatus* occurrence probability to environmental variables (Abbreviations as in Table 1).

The BRT model for *R. ciliatus* achieved optimal performance with a learning rate of 0.001, a bagging fraction of 0.5, a tree complexity of 5, and 3050 trees. The model had a mean deviance of 0.86, RMSE 0.26, training AUC 0.96, and cross-validation AUC 0.83. Water depth was the most influential predictor (16.50% contribution to the model), followed by nitrate nitrogen (12.30%). The predicted probability of occurrence was relatively high at depths ≥ 17 m and was also high when nitrate nitrogen concentrations ranged from 0.60 to 0.75 mg/L (Table 2 and Table 3, Figure 3 and Figure 8).

### 3.4. Spatio-Temporal Variation in the Predicted Occurrence Probability of Macrobenthic Species

To assess the spatio-temporal variation in macrobenthic species occurrence probability, *G. gurjanovae*, *S. scutata*, and *M. jedoensis* were selected based on their high frequency of occurrence. Using the constructed species distribution models, the summer occurrence probability distributions of these species were predicted annually from 2004 to 2023 across different regions of the Yellow River Estuary.

*G. gurjanovae* exhibited higher occurrence probabilities from 2009 to 2011 and 2016 to 2023. The total area with a high occurrence probability (≥0.5) was largest in 2017 (4452.73 km^2^), followed by 2010 (4107.54 km^2^). In 2008, 2013, and 2015, no areas had a high occurrence probability (≥0.5) (Figure S1). The predicted distribution of *S. scutata* was primarily concentrated in the northeastern and southeastern regions of the Yellow River Estuary, with relatively low occurrence probabilities near the estuary itself. The total area with a high occurrence probability reached its maximum in 2010 (4065.21 km^2^), followed by 2015 (3658.48 km^2^). In contrast, the smallest areas were recorded in 2009 (201.32 km^2^) and 2008 (191.96 km^2^) (Figure S2). For most years from 2004 to 2023 (excluding 2019), *M. jedoensis* exhibited consistently low occurrence probabilities. During 2004, 2010, 2012, and 2022, regions with high predicted occurrence probabilities were concentrated in the northeastern and southwestern parts of the Yellow River Estuary. The total area with a high occurrence probability (≥0.5) was largest in 2012 (2886.57 km^2^), followed by 2004 (2721.25 km^2^). In 2008, 2016, 2017, and 2023, no areas had a high occurrence probability (≥0.5) (Figure S3).

### 3.5. Influence of Yellow River Annual Discharge on the Distribution of Macrobenthic Species

**Figure 9:**
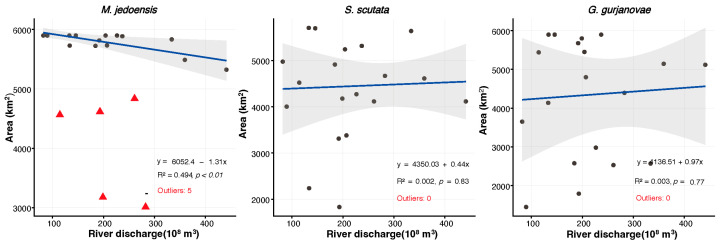
Interannual variations in the sea area associated with a 0–0.5 occurrence probability of dominant macro-benthic species and the Yellow River discharge. The fit line (solid blue), confidence interval (gray area), and outliers (red triangles) are displayed accordingly.

The Yellow River plays a crucial role in shaping the ecology of the Yellow River Estuary. This study applied the MM robust regression model to assess the relationship between annual discharge and the total area where *G. gurjanovae*, *S. scutata*, and *M. jedoensis* had a low occurrence probability (0–0.5). The results showed no significant correlation between annual discharge and the low-occurrence area of *G. gurjanovae* and *S. scutata*. However, a significant negative correlation was found for *M. jedoensis* (Figure 9). These findings indicate that higher annual Yellow River discharge reduces the total area where *M. jedoensis* had an occurrence probability between 0 and 0.5, explaining 46.46% of its variation.

## 4. Discussion

This study systematically investigated the distribution patterns and environmental drivers of dominant macrobenthic species in the Yellow River Estuary using a BRT model. It identified key environmental factors shaping species distributions and reproduced the spatio-temporal variations of dominant species over the past two decades. Five representative species were selected, including annelids (*G. gurjanovae*, *S. scutata*), bivalves (*M. jedoensis*, *T. fragilis*), and crustaceans (*R. ciliatus*). As essential components of the Yellow River Estuary ecosystem, the spatio-temporal distribution of these species in response to environmental changes can, to some extent, serve as an indicator of regional ecological conditions and the long-term dynamics of estuarine ecological processes.

### 4.1. Relationships Between Macrobenthic Fauna Distribution and Environmental Drivers

This study found that water depth was the most significant factor influencing the distribution of *S. scutata* and *R. ciliatus*. This relationship is likely driven by hydrodynamic variations along depth gradients [47,48,49,50]. Deeper areas provide more stable sediments with lower hydrodynamic disturbances, creating favorable habitats for benthic organisms [47]. Additionally, depth-driven variations in material exchange may also regulate species growth, reproduction, and settlement [50]. This finding aligns with previous studies which identified water depth as the key driver of benthic fauna distribution in the Yangtze River Estuary, central and southern Bohai Sea, and the Yellow and East China Seas, respectively [51,52]. No significant correlation was observed between the annual discharge of the Yellow River and the area of low-occurrence probability zones for *S. scutata*. This may be due to two factors: first, *S. scutata* is a pollution-tolerant species with high resilience to environmental disturbances [53]; second, it primarily inhabits deeper waters, where river discharge fluctuations may have minimal influence. Further correlation analyses, considering specific environmental contexts and temporal variations, may provide deeper insights into how discharge fluctuations shape the spatio-temporal distribution of this species.

Ammonia nitrogen, salinity, and nitrate nitrogen were identified as key determinants of *G. gurjanovae* distribution. This species exhibited a preference for lower ammonia nitrogen and nitrate nitrogen concentrations, with habitat suitability increasing as concentrations declined. It also favored high-salinity environments (≥30‰). Cai et al. [54] reported that eutrophication significantly reshapes benthic community structure and alters dominant species composition in Bohai Bay. In this study, the dominance of *G. gurjanovae* increased markedly from 2009 onward. According to Wu et al. [55], inorganic nitrogen concentrations peaked in 2006 in the Yellow River Estuary, then gradually declined, reaching approximately 30 μmol/L by 2009, and remained below this threshold until 2023. This reduction in inorganic nitrogen likely enhanced habitat suitability for *G. gurjanovae* in the region. As a relatively sensitive species in the AZTI’s Marine Biotic Index (AMBI) system [53], *G. gurjanovae* is susceptible to mild pollution and anthropogenic disturbances, suggesting that improved water quality may benefit its survival. No significant correlation was observed between the annual discharge of the Yellow River and the area of low-occurrence probability zones for *G. gurjanovae*. Given that the Yellow River is the primary source of nutrients and freshwater in this marine system [55,56], this result does not necessarily preclude an influence of river discharge on species distribution. Instead, the weak correlation likely reflects the dominant role of nutrient concentration fluctuations, rather than discharge volume alone, in regulating *G. gurjanovae* distribution.

*M. jedoensis* is a typical marine estuarine benthic species [57] that responds strongly to sediment grain size and salinity variations. Unlike *S. scutata* and *G. gurjanovae*, which prefer fine silt-rich sediments, *M. jedoensis* is less adapted to environments with minimal sand content. Yuan et al. [58] also found that bivalves are highly sensitive to sediment grain size, which significantly influences their spatial distribution along the coast of Qingdao. The area of low-occurrence probability zones for *M. jedoensis* is negatively correlated with the Yellow River discharge, suggesting that higher discharge expands the suitable habitat (occurrence probability > 0.5). This trend aligns with the positive relationship between species occurrence probability and chl-a concentration. The underlying reason may be that increased Yellow River discharge enhances phytoplankton productivity and terrestrial organic input [57,59], thereby providing more food for *M. jedoensis* and improving its habitat suitability. Similar patterns have been reported in the Yangtze and Pearl River estuaries, where river discharge significantly influences the distribution of macrobenthic fauna [60,61], highlighting the broader role of riverine input in shaping estuarine benthic ecosystems.

The distribution of *T. fragilis* is strongly affected by water temperature, with its occurrence probability dropping significantly above 25 °C. This is likely due to the species’ physiological and ecological traits [62], as temperature changes strongly influence metabolic rates and life cycle rhythms in benthic organisms, which in turn shape their spatial distribution and population dynamics [63]. These findings suggest that climate-driven warming may profoundly affect the distribution of *T. fragilis*.

Spatially, the northeastern Yellow River Estuary exhibited higher habitat suitability for *S. scutata* and *M. jedoensis* in most study years. As the primary outflow channel of the Yellow River, this region receives significant terrestrial input, which enhances the occurrence probability of these two benthic species. In years when *G. gurjanovae* had a higher occurrence probability (>0.5) across large areas (2009, 2011, 2016, 2018, 2022, and 2023), its abundance was significantly lower in the northeastern estuary. This region, where Yellow River discharge enters the sea, experiences strong plume influence [25]. This pattern is consistent with the correlation analysis between Yellow River discharge and the unsuitable habitat area.

Fu et al. [64] identified salinity and sediment type as the primary factors shaping macrozoobenthos distribution patterns in the northern and southern intertidal zones of the Yellow River Delta. In this study, salinity was not a major determinant of macrozoobenthos distribution, except for *G. gurjanovae*. Likewise, Li et al. [23] found that salinity variations had no significant impact on the biomass and density of most benthic species across the Yellow River Estuary, although these species generally preferred deeper waters and higher salinity levels. Notably, the factors influencing species distribution in the Yellow River Estuary are dynamic, responding to climate change and human activities. During water-sediment regulation periods, the large influx of freshwater and terrestrial materials may substantially alter benthic organism distribution, particularly in the northeastern estuary and river mouth region [23]. Consequently, some species may exhibit environmental responses that differ from those observed in this study.

In this study, ammonia nitrogen had a strong influence on the distribution of *G. gurjanovae*, *T. fragilis*, and *R. ciliatus*; all dominant benthic species had higher occurrence probabilities at lower NH_4_^+^-N concentrations. Their occurrence probability notably increased when NH_4_^+^-N dropped below 0.1 mg/L. In the Yellow River Estuary, NH_4_^+^-N concentrations ranged from 0.005 to 0.26 mg/L, averaging 0.060 mg/L. Lowering NH_4_^+^-N concentrations in this region may probably improve the habitat suitability for most dominant benthic species.

### 4.2. Model Limitations and Optimization Directions

In this study, the training AUC values for all macrobenthic species exceeded 0.95, while the predictive AUC values all exceeded 0.70, indicating that the model achieved relatively high predictive accuracy. However, in the analysis of key influencing factors, no single factor exhibited a substantially greater influence on species distribution than others. This may suggest that some critical environmental factors influencing the distribution of large benthic animals remain unidentified. For instance, food availability and hydrodynamic forces, which are recognized as key determinants of marine species distribution [65], were not included as variables in the model during training. Alternatively, this could be attributed to the relatively small spatial scale of the Yellow River Estuary, where the overall marine ecological environment is relatively homogeneous, leading to a relatively uniform distribution of large benthic animals. Additionally, it is worth noting that this study only considered monitoring data from the spring and summer seasons, which may have limited the ability to fully capture the habitat preferences of dominant benthic species in response to environmental factors. Incorporating dietary preferences, physiological characteristics, and hydrodynamic factors, along with expanding the study area and improving seasonal-scale monitoring data to increase data diversity and volume, may facilitate the identification of key environmental drivers influencing species distribution and improve model predictive performance.

### 4.3. Implications for Management

In this study, inorganic nitrogen, sediment sand content, and water temperature were identified as key factors influencing the distribution of large benthic organisms. Human activities, including terrestrial pollution discharge and water-sediment regulation in the Yellow River, significantly alter inorganic nitrogen concentrations and sediment characteristics in marine environments. Additionally, ongoing climate warming may further intensify these environmental changes. Our findings suggest that both anthropogenic activities and climate change may have a significant influence on the distribution of macrobenthic fauna. Because ammonia nitrogen strongly influences the distribution of most benthic organisms, it may serve as a critical indicator of habitat suitability for macrobenthic communities. As terrestrial inputs from the Yellow River constitute a major source of ammonia nitrogen in the Yellow River Estuary [56], reducing ammonia nitrogen loading from the watershed could lower ammonia nitrogen concentrations in the estuarine system, thereby improving habitat conditions for macrobenthic organisms. Furthermore, considering the contrasting effects of Yellow River discharge and nutrient input on different species, minimizing human-induced fluctuations in river flow is essential for maintaining the stability of macrobenthic community structure within the estuarine ecosystem.

Due to the absence of future environmental data, this model can only reconstruct the historical distribution of dominant benthic species. It cannot predict their future distribution under projected environmental scenarios. To overcome this limitation, future studies could use ecological dynamic models to predict changes in marine environmental variables [66]. The terrestrial watershed models [67], such as those for the Yellow River, can provide essential terrestrial input data to drive ecological dynamic models. This approach could facilitate projections of future macrobenthic species distributions. Such projections may in turn offer valuable technical and theoretical support for the regulation of terrestrial inputs and the conservation of marine ecosystems.

## 5. Conclusions

Based on historical monitoring data from the Yellow River Estuary, this study applied the Boosted Regression Tree model to develop species distribution models for five dominant macrobenthic species. The models predicted the spatial probability distribution of these species over the past two decades in the estuary. Water depth, ammonia nitrogen, water temperature, and sand content of sediment were identified as the primary determinants of their distribution.

Macrobenthic organisms exhibited distinct response patterns to environmental changes in the estuary, which may be linked to shifts in their key habitats. Most species responded similarly to ammonia nitrogen, providing a key reference for benthic habitat management and optimization. The Yellow River played a crucial role in shaping the distribution of dominant benthic species, particularly in the areas influenced by its plume. However, temporal variations in nutrient concentrations may lead to fluctuating impacts of the river on macrobenthic species distribution.

Enhancing seasonal coverage of monitoring data and integrating hydrodynamic and food-resource variables may further improve model accuracy. Future research that integrates coupled hydrodynamic–ecological models with terrestrial watershed models could enhance the predictive accuracy of species distribution patterns, thereby offering more robust theoretical and technical support for ecological conservation and management in the Yellow River Estuary.

## Figures and Tables

**Table 1:** Predictor variables selected for boosted regression tree models of macrobenthic species in Yellow River estuary.

Target Species	Predictor Variables
*S. scutata*, *T. fragilis*, *R. ciliatus*	WD, DO, SA, RP, NIN, NAN, AN, chl-a, N/P, pH, WT, ACS
*M. jedoensis*	WD, DO, SA, RP, NIN, NAN, AN, chl-a, N/P, WT, ACS
*G. gurjanovae*	WD, DO, SA, RP, NIN, NAN, AN, chl-a, N/P, WT, ACS, ICS

Note: WD: water depth; DO: dissolved oxygen; SA: salinity; RP: reactive phosphate; NIN: nitrite nitrogen; NAN: nitrate nitrogen; AN: ammonia nitrogen; chl-a: chlorophyll-a; N/P: nitrogen-to-phosphorus ratio; WT: water temperature; ACS: sand content of sediment; ICS: silt content of sediment.

**Table 2:** BRT model parameters for macrobenthic species.

Species	LR	TC	No. of Trees	BF
*G. gurjanovae*	0.0005	5	1800	0.5
*S. scutata*	0.005	15	3700	0.5
*M. jedoensis*	0.001	5	4400	0.5
*T. fragilis*	0.001	5	4020	0.5
*R. ciliatus*	0.001	5	3050	0.5

Note: LR: learning rate; TC: tree complexity; No.: number; BF: bagging fraction.

**Table 3:** Performance validation of BRT models for macrobenthic species.

Species	Training AUC	CV AUC	RMSE	MAE	MD
*G. gurjanovae*	0.96	0.73	0.35	0.32	1.18
*S. scutata*	0.97	0.75	0.34	0.32	1.11
*M. jedoensis*	0.96	0.71	0.36	0.33	1.19
*T. fragilis*	0.99	0.78	0.25	0.18	0.91
*R. ciliatus*	0.96	0.83	0.26	0.17	0.86

Note: AUC: area under the receiver operating characteristic curve; CV: cross-validation; RMSE: root mean squared error; MAE: mean absolute error; MD: mean deviance.

## Data Availability

The datasets generated and/or analyzed during the current study are available from the corresponding author on reasonable request.

## Supplementary Materials

The following supporting information can be downloaded at: https://www.mdpi.com/article/10.3390/biology14121732/s1, Table S1. Statistical summary of model variables. Figure S1. Spatio-temporal distribution of Glycinde gurjanovae occurrence probability from 2004 to 2023. Figure S2. Spatio-temporal distribution of Sternaspis scutata occurrence probability from 2004 to 2023. Figure S3. Spatio-temporal distribution of Moerella jedoensis occurrence probability from 2004 to 2023.
