# Supplementary material for: Modeling Dominant Macrobenthic Species Distribution and Predicting Potential Habitats in the Yellow River Estuary, China
Source: Biology (Basel). 2025 Dec 3;14(12):1732. doi: 10.3390/biology14121732 (PMC12730213; doi:10.3390/biology14121732)
Supplement: Supplementary file 1 [file biology-14-01732-s001.zip › biology-3965476-supplementary.pdf]

Supplement materials

Table S1. Statistical summary of model variables

Variable	VIF	Mean	SD	Range	N
WD (m)	1.20	10.64	5.17	1.00–19.00	608
pH	1.38	8.21	0.07	7.96–8.41	608
WT	1.05	23.14	5.06	10.32-30.95	608
DO (mg/L)	1.43	8.82	1.69	4.55–12.11	608
SA (‰)	1.72	28.75	1.91	12.93–32.32	608
RP (mg/L)	1.90	0.01	0.01	0.0009–0.045	608
AN (mg/L)	1.34	0.06	0.04	0.005–0.26	608
NAN (mg/L)	1.89	0.47	0.22	0.12–1.40	608
NIN (mg/L)	1.69	0.02	0.03	0.0032–0.23	608
Chl-a (μg/L)	1.32	3.33	2.57	0.74–15.36	608
ACS (%)	1.76	25.55	16.98	1.10–65.40	608
ICS (%)	1.87	63.8	17.32	32.30–93.50	608
OCS (mg/g)	1.20	0.22	0.123	0.016–0.827	608
N/P	1.58	78.40	84.70	4.14-818.75	608
WD (m)	1.20	10.64	5.17	1.00–19.00	608

Note: VIF: variance inflation factor; SD: standard deviation; N: number; WD: water depth; WT: water temperature; DO: dissolved ox-ygen; SA: salinity; RP: reactive phosphate; AN: ammonia nitrogen; NAN: nitrate nitrogen; NIN: nitrite nitrogen; Chl-a: chlorophyll-a; ACS: sand content of sediment; ICS: silt content of sediment; OCS: Organic carbon of sediment; N/P: nitrogen-to-phosphorus ratio.

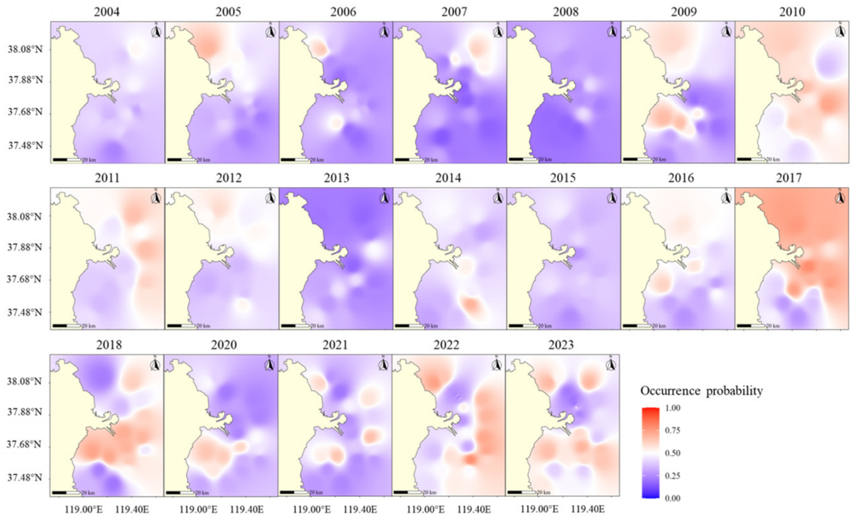

Figure S1. Spatio-temporal distribution of *Glycinde gurjanovae* occurrence probability from 2004 to 2023

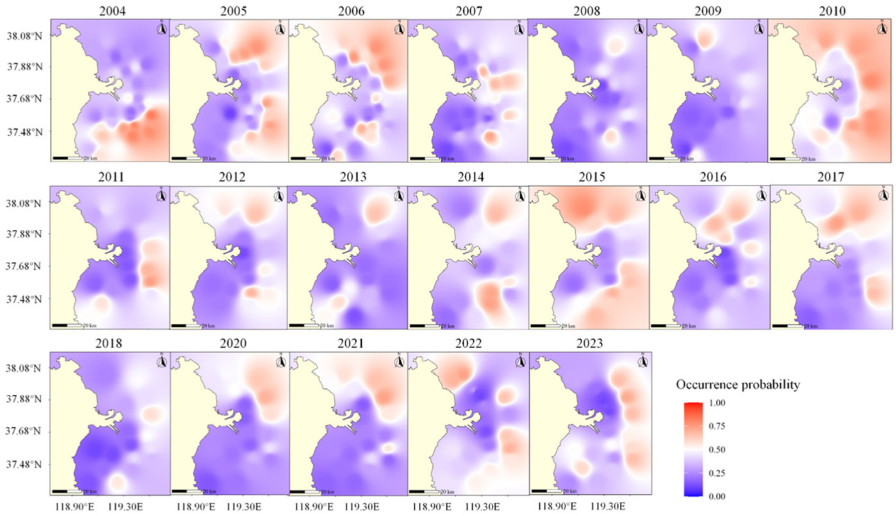

Figure S2. Spatio-temporal distribution of *Sternaspis scutata* occurrence probability from 2004 to 2023

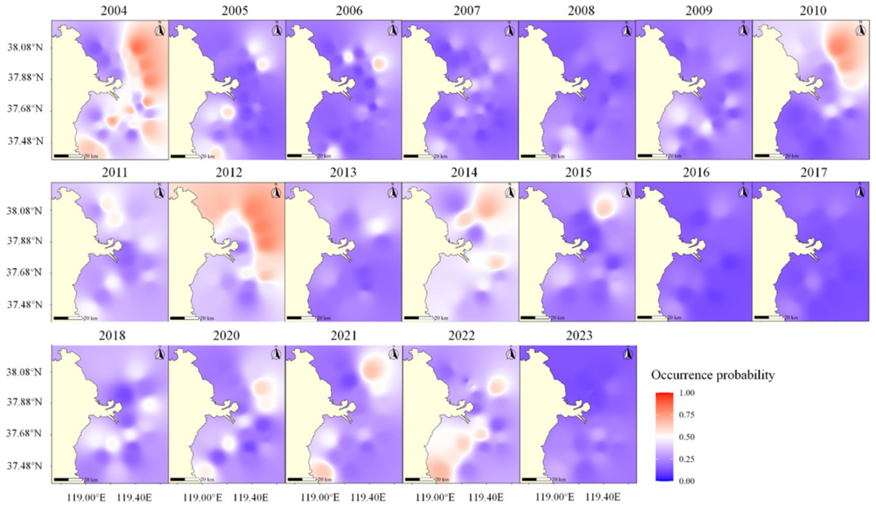

Figure S3. Spatio-temporal distribution of *Moerella jedoensis* occurrence probability from 2004 to 2023
